# Cost-Effectiveness Analysis of BCG Vaccination against Tuberculosis in Indonesia: A Model-Based Study

**DOI:** 10.3390/vaccines8040707

**Published:** 2020-11-26

**Authors:** Afifah Machlaurin, Franklin Christiaan Karel Dolk, Didik Setiawan, Tjipke Sytse van der Werf, Maarten J. Postma

**Affiliations:** 1 Department of Health Sciences, University Medical Center Groningen, University of Groningen, 9713 AV Groningen, The Netherlands; m.j.postma@rug.nl; 2 Department of Clinical and Community Pharmacy, Universitas Jember, Jember 68121, Indonesia; 3 Unit of Pharmaco-Therapy, Epidemiology & Economics (PTE2), Department of Pharmacy, University of Groningen, 9713 AV Groningen, The Netherlands; f.c.k.dolk@rug.nl; 4 Faculty of Pharmacy, Universitas Muhammadiyah Purwokerto, Purwokerto 53182, Indonesia; d.didiksetiawan@gmail.com; 5 Department of Pulmonary Diseases & Tuberculosis, University Medical Center Groningen, 9713 AV Groningen, The Netherlands; t.s.van.der.werf@umcg.nl; 6 Department of Economics, Econometrics & Finance, Faculty of Economics & Business, University of Groningen, 9747 AE Groningen, The Netherlands; 7 Department of Pharmacology & Therapy, Universitas Airlangga, Surabaya 60132, Indonesia; 8 Center of Excellence in Higher Education for Pharmaceutical Care Innovation, Universitas Padjadjaran, Bandung 45363, Indonesia

**Keywords:** cost-effectiveness analysis, BCG, vaccine, tuberculosis

## Abstract

Bacillus Calmette–Guerin (BCG), the only available vaccine for tuberculosis (TB), has been applied for decades. The Indonesian government recently introduced a national TB disease control programme that includes several action plans, notably enhanced vaccination coverage, which can be strengthened through underpinning its favourable cost-effectiveness. We designed a Markov model to assess the cost-effectiveness of Indonesia’s current BCG vaccination programme. Incremental cost-effectiveness ratios (ICERs) were evaluated from the perspectives of both society and healthcare. The robustness of the analysis was confirmed through univariate and probabilistic sensitivity analysis (PSA). Using epidemiological data compiled for Indonesia, BCG vaccination at a price US$14 was estimated to be a cost-effective strategy in controlling TB disease. From societal and healthcare perspectives, ICERs were US$104 and US$112 per quality-adjusted life years (QALYs), respectively. The results were robust for variations of most variables in the univariate analysis. Notably, the vaccine’s effectiveness regarding disease protection, vaccination costs, and case detection rates were key drivers for cost-effectiveness. The PSA results indicated that vaccination was cost-effective even at US$175 threshold in 95% of cases, approximating the monthly GDP per capita. Our findings suggest that this strategy was highly cost-effective and merits prioritization and extension within the national TB programme. Our results may be relevant for other high endemic low- and middle-income countries.

## 1. Introduction

Tuberculosis (TB) is the leading cause of death from infectious diseases, accounting for the largest burden in low- and middle-income countries. In 2017, there were 845,000 new cases of TB in Indonesia, which has the third highest TB burden after India and China in terms of the incidence per year [1]. The WHO has set a target of achieving a TB-free world by 2035, with the aim of reducing the TB-incidence to below 10/100,000 people. Accordingly, by 2020, the TB-incidence should fall below 85/100,000 and the TB mortality rate should decline to 10% or less [2]. However, the TB incidence in Indonesia remains well above this target at 316/100,000 people, and the TB fatality rate was still 12% in 2017 [3].

In 2016, the Indonesian government framed a national programme for controlling TB through several action plans: health promotion, TB surveillance, controlling risk factors, finding and treating new TB cases, extending vaccinations, and prophylactic treatment of latent TB [4]. As part of cumulating strategies within the national programme, Bacillus Calmette–Guerin (BCG) vaccination has been administered since 1956 [5]. In 2018, Indonesia’s national TB budget was US$294 million, of which just 16% was supported through external funds, such as the Global Fund. In light of its policy of reducing dependency on external grants, Indonesia will probably have to expand its budgetary allocation for the TB programme in the future [6]. BCG vaccinations can help by providing protection against meningeal and miliary TB in infants [7,8]. However, in countries located near the equator, including Indonesia, the efficacy of the BCG vaccine is extremely variable within the adult population [8,9,10], while the efficacy is more stable and higher in low-incidence and low-mycobacterial TB-exposure regions at higher latitudes [9,10]. The vaccine efficacy (VE) also appears to wane over a time period of 10 to 40 years [10,11,12,13]. There have been promising steps in the development of new tuberculosis vaccines in the last decade, with 12 candidates entering clinical trial phase [14]. Yet, there is not yet any vaccine potentially replacing BCG available in the market. While awaiting the introduction of new vaccines that provide better protection, BCG vaccination is warranted, and decision-makers should give more attention and allocate an adequate budget to such TB vaccination programmes.

Economic evaluations of universal BCG vaccination given at birth in high TB-incidence countries are rare [15]. Only one economic evaluation was conducted in Indonesia in 1980, in which independent administration of the BCG vaccine was not considered cost-effective at that time because of the high supply chain and delivery costs. Still, its administration within a combined immunization programme, such as with the diphtheria-pertussis-tetanus toxoid (DPTT) vaccine, was deemed justifiable [16]. However, a joint vaccination strategy is not applicable to the current context, as the prevailing BCG vaccination strategy in Indonesia is to vaccinate infants within the first month of their birth, whereas DPTT vaccination occurs later [17]. While in some countries with low TB incidence, BCG vaccination is not performed or given selectively at high risk groups, continuation of the universal vaccination in some countries had risen a question in terms of its health economic impacts [15]. Considering Indonesia’s limited healthcare resources, a re-evaluation of the cost-effectiveness of the BCG vaccination is pertinent to inform decision makers about the relative importance of this strategy within the TB control programme that could potentially warrant increased coverage if considered cost-effective. The aim of this study was thus to assess the cost-effectiveness of Indonesia’s current BCG vaccination strategy and to provide recommendations regarding the next developmental phase of the vaccination programme.

## 2. Materials and Methods

### 2.1. Model Settings

A static Markov model was developed to assess the cost-effectiveness of Indonesia’s current BCG vaccination programme. People in each compartment can move in or out to other compartments with specific probabilities (Table 1). The model did not explicitly consider any transmission between population and compartments. We applied our single-cohort model to predict the costs and health impacts on 4,900,000 infants as our initial “healthy” population, reflecting the 2017 Indonesian birth cohort [5]. A lifetime horizon of 70 years was applied, as grossly reflecting the expected life span in Indonesia. Annual cycles were applied as time steps in the Markov model. For the base case, the vaccine uptake was set at the current national level of 87% [5]. We used the Microsoft Excel 2016 programme to run the model and perform the sensitivity analysis. A 3% discount rate, based on a WHO universal guideline, was applied for both the costs and the outcomes [18]. Table 1 shows the input parameters for the analysis. We validated the model by comparing the TB incidence derived from the predicted simulation with that in the observed dataset used for the input parameters. We calibrated the model-predicted TB prevalence on that obtained from local data in 2018 [5].

The incremental cost-effectiveness ratio (ICER) was calculated as the incremental cost per incremental quality-adjusted life years (QALY) gained for a strategy of implementing universal vaccinations compared with one entailing no vaccinations. The cost-effectiveness of these strategies was evaluated against the per capita GDP, applying a threshold at triple the GDP per capita. The strategy was considered cost-effective if the ICER fell below the willingness-to-pay as the threshold. We performed univariate and multivariate (probabilistic) sensitivity analyses (PSA). The univariate analysis was performed to determine the key drivers of the cost-effectiveness analysis by varying the base case value with lower and upper values of each parameter. We opted to use confidence intervals (CIs) in the sensitivity analysis; when these were unavailable, we used ±25% deviations. The PSA was performed by running 1000 iterations in a Monte Carlo simulation. The results of this simulation were presented as cost-effectiveness acceptability curves, using the threshold values to predict the probability of the strategy being cost-effective.

### 2.2. Natural History of TB

**Figure 1:**
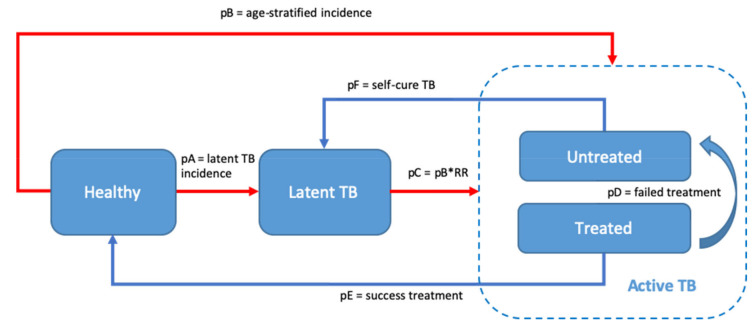
A model depicting the natural course of tuberculosis (TB). This model was based on the age-stratified incidence of TB and implicitly included the mortality rate at every stage. The red line is affected by the vaccine effectiveness. pA = probability of infection reflected as latent TB incidence, pB = probability of fast activation reflected as age-stratified active TB incidence, pC = probability of slow progression calculated by multiplying TB incidence with relative risk (RR) of developing TB disease in latent TB population, pD = probability of treatment failure, pE = probability of successful treatment, pF = probability of self-cure. Detailed transition probabilities were described in Table 1.

Some natural TB pathways have been proposed in modelling studies [30,31,32]. In this study, we modified these models as shown in Figure 1. Specifically, we assumed that individuals who were infected with *Mycobacterium tuberculosis* (*M. tb*) after birth could either directly develop (active) TB disease or they could be infected without developing the disease (latent TB infection). There is evidence of protection against the progression of latent TB to active TB [21]. Therefore, the group with latent TB was assigned a lower risk of developing active TB compared with uninfected or healthy individuals. Individuals with active TB were categorized either as drug-susceptible TB (DSTB) patients or as multidrug-resistant TB (MDRTB) patients, whose TB may or may not have been detected and treated. We assumed that successful treatment entailed sterilization of all *M. tb*, resulting in a full return to health. Failed treatment was deemed as similar to a relapse, as a result of which patients returned to the group with (untreated) active TB. Undetected cases were assumed to progress, as untreated patients who continued to be chronic TB patients would either recover and become latent TB patients or their disease would advance, leading to their deaths. Applying an exponential formula, we calculated the annual mortality and self-cure rates of untreated TB patients using the estimate of 10 years’ case fatality rate of smear-negative TB patients, which was derived from a systematic review that included 16 cohort studies [25]. The mortality rate of the healthy population followed the natural age-stratified death risk for the Indonesian population [33].

### 2.3. Incidence of Tuberculosis Infection

We calculated the age-stratified incidence relating to the probability of contracting active TB from the total number of new and relapsed cases in each age group within the total population. The number of cases was derived from a WHO report on the TB burden in Indonesia in 2017 [22]. For the total population in each group, we used demographic projection data for the Indonesian population issued by the Indonesian Central Bureau of Statistics in 2017 (Badan Pusat Statistik; BPS) [19]. Using the same sources, we calculated the risk of infection and the probability of developing latent TB. The latter figure was estimated by dividing the number of latent TB cases, defined as the number of children under the age of 5 years who are exposed to TB patients within their households and are eligible for preventive therapy, by the population size of children under the age of 5 years. The mortality rate for the latent TB group was derived from an observational study conducted by Miller et al., in which the death rate for the latent TB group without a history of TB disease prior to death was reportedly 3.1% [24].

TB incidences within the vaccinated and unvaccinated groups could not be determined directly using the incidence rates reported in the WHO report [22], as the data were derived from the current Indonesian situation in which BCG vaccination has been applied for over a long period. A formula incorporating the observed incidence, vaccine coverage, and vaccine effectiveness was used to calculate the TB risk within the unvaccinated group [34]. The TB incidence within the vaccinated group was accordingly calculated using a universal formula for vaccine effectiveness.

(1) $$I_{unvaccinated}=\;\frac{I_{observed}}{1-\left(ve*vc\right)}$$

(2) $$I_{vaccinated}=\left(1-ve\right)*I_{unvaccinated}$$

where *vc* denotes vaccine coverage, *ve* denotes vaccine effectiveness, *I_observed_* denotes the observed TB incidence, and *I_vaccinated_* and *I_unvaccinated_* denote the TB incidence among vaccinated and unvaccinated individuals, respectively.

### 2.4. Characteristics of the BCG Vaccine

**Figure 2:**
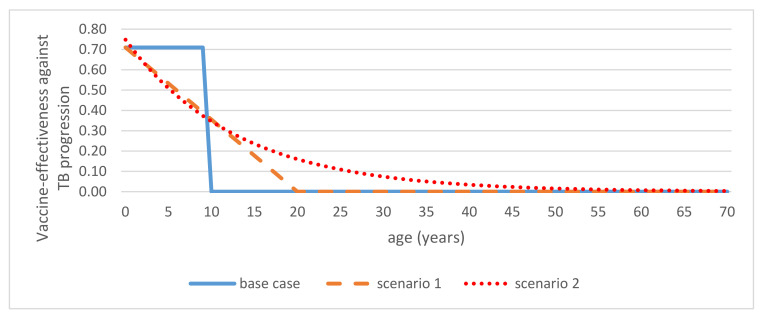
Waning effect scenarios for the effectiveness of the Bacillus Calmette–Guerin (BCG) vaccine against tuberculosis (TB) progression. Three different periods of waning protections were considered: A 10 year-only protection (base case), a linear waning effect over 20 years (scenario 1), and an exponential waning effect (scenario 2). The same waning scenarios were applied to other values of vaccine efficacy (VE).

Cross-country evidence on the effectiveness of the BCG vaccine is inconsistent, with increased effectiveness being associated especially with high-latitude countries. Relatively few studies have assessed the effectiveness of the BCG vaccine in countries with high incidences of TB, specifically Indonesia. To tackle this issue, instead of using one specific estimate from one study in Indonesia, we applied estimates of the BCG vaccine’s effectiveness obtained from a meta-analysis study, encompassing 14 studies worldwide, including one from Indonesia. These estimates were 27% (risk ratio: 0.73, CI: 0.61–0.87) and 71% (risk ratio: 0.29, CI: 0.15–0.58) for latent TB infection and active TB, respectively. Additionally, this study reported that the vaccine evidently provided protection against progression from an infected state to active disease estimated at 58% (risk ratio: 0.42, CI: 0.23–0.77) [9]. We conservatively assumed that the BCG vaccine provided protection for a 10-year period and no protection thereafter in the base case [10]. For the base case, the national vaccine coverage was 87% [5]. In addition, we considered some waning scenarios as presented in Figure 2. First, we assumed that the vaccine effectiveness would wane linearly over a 20-year period. Accordingly, we estimated that the average protection would be comparable to that assumed in the base-case scenario. Second, we assumed that the vaccine’s effectiveness would wane exponentially over time. The fitting formula is shown in the Supplementary Materials.

### 2.5. Treatment Outcomes

Active TB patients could have either DSTB or MDRTB and would receive the relevant treatment associated with a set of outcomes. In our model, we applied the case detection rate as the probability for TB patients who were eligible to be treated and applied the treatment outcomes provided by the WHO for the specific Indonesian dataset [22]. Four outcomes were included, categorized as: success, failure, death, and unknown outcomes or cases lost to follow-up. We assumed that the patients lost to follow-up would remain in the active TB treatment phase.

### 2.6. Utilities

The outcomes were measured as life years gained (LYGs) as well as quality-adjusted life years (QALYs). LYGs were calculated by summing up the number of people alive in each compartment in each cycle. We ran 70 cycles aligned with the standard life expectancy at birth. QALYs were calculated by multiplying the people alive in each compartment with a specific utility factor (Table 1). We applied the same utility of 0.68 for active TB patients with or without treatment. However, we applied a higher utility estimate of 0.82 for latent TB patients compared with active TB patients. We assumed that the healthy population enjoyed full utility (valued at 1.0). These estimates are shown in Table 1.

### 2.7. Costs

The analysis was performed from both the societal and healthcare perspectives. Whereas only the cost of vaccination and medical treatment were considered in the latter analysis, the loss of productivity was also considered in the former analysis. We applied the cost of vaccination only in the first year of the cycle at a certain coverage for the vaccination strategy. The medical cost incurred by the patients in active TB compartments was calculated by multiplying the number of patients with the treatment cost for DSTB and MDRTB accordingly, while untreated patients were assumed to incur no medical cost. We calculated the cost of work loss during DSTB and MDRTB treatments by multiplying the days lost by the age-stratified daily minimum wage in Indonesia [35]. All costs were discounted annually at 3%. The estimates of treatment costs were derived from the WHO report on the utilization and expenditure of TB treatment resources [27] using the Indonesia dataset for 2017. Costs incurred through lost productivity were derived from costing studies about the economic burden of TB in Indonesia and were estimated respectively as 25 and 102 work days lost by DSTB and MDRTB patients as a result of illness [6,36]. We assumed that the productivity loss would only burden patients aged between 15 and 60 years, which is the age range for productive persons in Indonesia. We considered productivity losses incurred by the parents of children (aged 0–15 years), based on the assumption that at least one caregiver would be absent from work to take care of the sick child. We assumed that the untreated patients did not incur any direct medical costs or productivity losses. The cost of the BCG vaccination package was obtained from the pricelists of private Indonesian healthcare providers [26]. Therefore, the assumed cost included the vaccine price as well as the cost of administration. All of the referential pre-2017 costs included in the analysis were converted to US$ prices in 2018 by applying purchasing power parities.

## 3. Results

### 3.1. Base-Case Analysis

**Figure 3:**
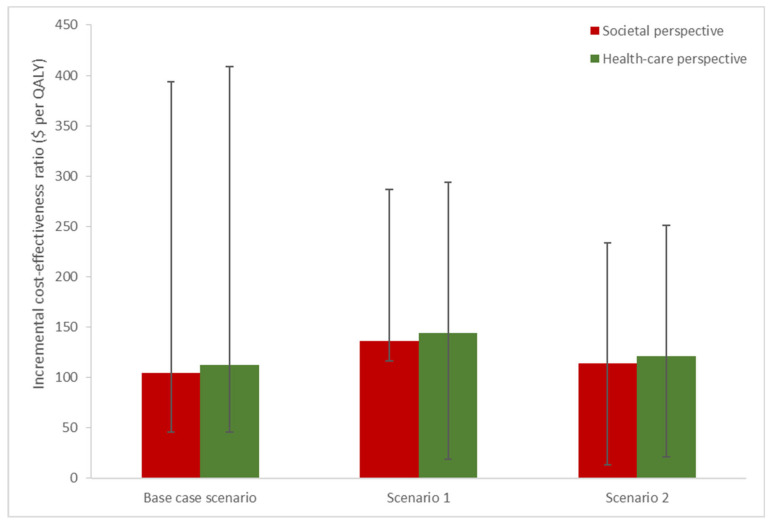
The incremental cost-effectiveness ratios (ICERs) of vaccination strategies compared with a no-vaccination strategy. The bar heights show the ICERs for a base assumption of 71% vaccine effectiveness against TB, and the error bars indicate the lower (42%) and upper (85%) ranges of cost-effectiveness. Three different waning protection scenarios were applied: protection for the first 10 years (base case), a linear waning effect of 20 years (scenario 1), and an exponential waning effect (scenario 2). Red and green bars show the societal and health-care perspective, respectively.

Using a static Markov model design, we applied the model for the Indonesian national birth cohort in 2017, comprising 4,900,000 infants. Applying a base-case assumption of protection for the first 10 years after birth, we determined that a BCG vaccination strategy costing around US$57 million at an uptake level of 87% would yield 488,592 QALYs and would save around US$51 million and US$55 million from the societal and healthcare, respectively (Table 2). This vaccination strategy yielded ICER values of US$104/QALY and US$112/QALY from the perspectives of society and healthcare, respectively. The application of a linear waning effect of 20 years in scenario 1 resulted in an almost twofold increase in the respective ICERs for society and healthcare perspective (US$226/QALY and US$233/QALY). However, the ICERs did not differ significantly when an exponential waning effect was applied in scenario 2 (US$113/QALY and US$121/QALYs for society and healthcare perspective, respectively). Taken together the ICERs, the above vaccination strategy can be considered highly cost-effective because all of the ICERs remained well below the Indonesian one GDP per capita (US$3847) figure in 2018, as depicted in Figure 3.

Based on the internal and external model calibration, our model was considered valid. The projection of the model prediction followed an identical trend of the observed data. Figures are shown in Supplementary Materials.

### 3.2. Univariate Sensitivity Analysis

**Figure 4:**
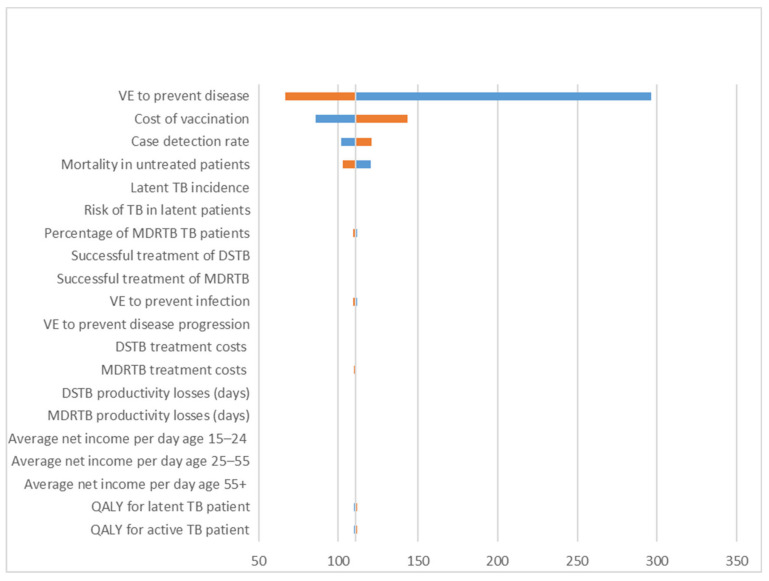
A tornado diagram of the univariate analysis. VE = vaccine effectiveness, TB = tuberculosis, DSTB = drug-susceptible tuberculosis, MDRTB = multidrug-resistance tuberculosis, QALY = quality-adjusted life years.

Figure 4 depicts a tornado diagram for varying model input parameters that influenced the ICERs. The VE for disease prevention, the vaccination costs, the case detection rate, and the natural case fatality rate of untreated TB were the four most important drivers in the analysis, with other factors minimally influencing the ICERs.

### 3.3. Probabilistic Sensitivity Analysis

**Figure 5:**
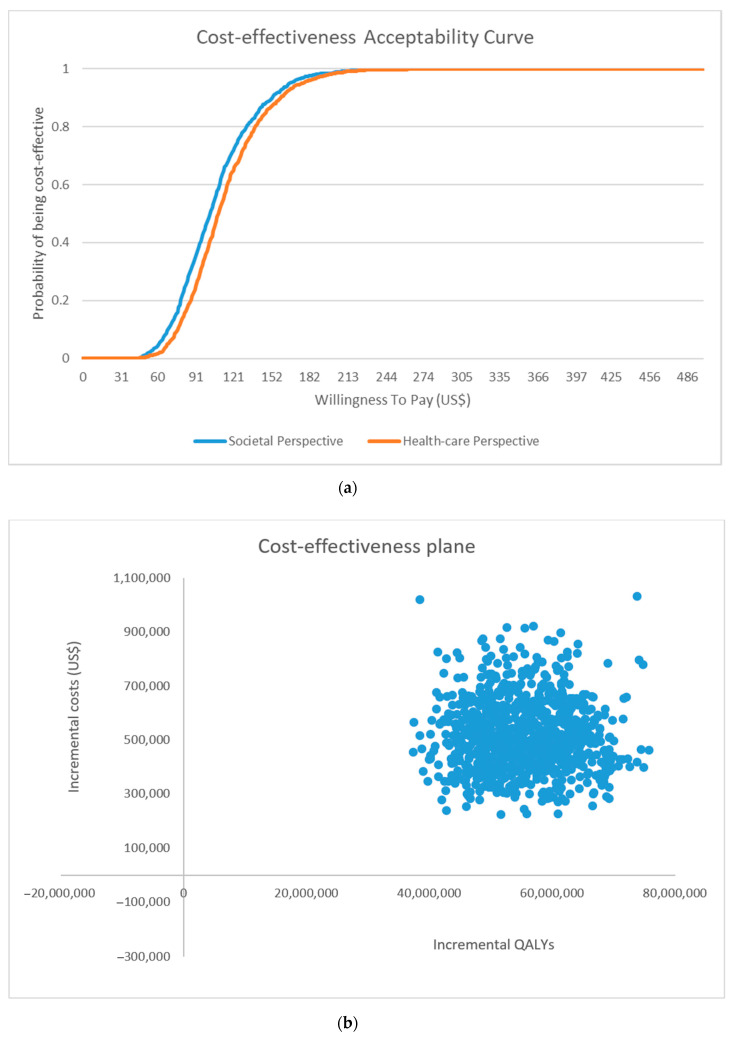
(**a**) Cost-effectiveness acceptability curves and (**b**) Cost-effectiveness plane of health-care perspective obtained from the probabilistic sensitivity analysis. QALYs = quality-adjusted life years.

Figure 5 shows the probability of the strategy being cost-effective according to different levels of willingness-to-pay threshold values. In the base case, BCG vaccination, priced at US$14, was 100% cost-effective at a threshold value of one GDP per capita, with a 95% probability of being cost-effective at US$175, which approximates the monthly GDP per capita.

## 4. Discussion

By applying TB epidemiology data and age-stratified TB incidence rates for Indonesian context, we analysed its universal BCG vaccination, representing a range of BCG efficacies against TB infection, disease, and progression and applying various waning protection and duration. The results of our static modelling have indicated that a BCG vaccination strategy was highly cost-effective compared with a strategy of no vaccinations. Priced at US$14 per vaccination per infant, the vaccine could prevent 49,713 TB cases and 7598 TB deaths (Table 2). Moreover, from both a healthcare payer and societal perspective, the strategy entailed a threshold that was well below one GDP per capita per QALY, remaining below the monthly GDP per capita.

The strategy’s cost-effectiveness was found to be robust for most of the input parameters in the one-way sensitivity analysis. Concurring with another health economic studies conducted on BCG vaccination [15], this study showed that vaccine efficacy against TB disease was the most influential variable in the cost-effectiveness analysis. Despite the fact that BCG vaccination was introduced several decades ago, it has remained controversial in terms of its efficacy. One reason for these inconsistencies could be the differing rates of prior exposure to non-tuberculosis mycobacteria (NTM) [8]. In particular, individuals in low-latitude areas and older children are more prone to NTM exposure [37]. Given the conflicting evidence, we applied a relatively conservative assumption in our model. Our analysis of the VE profile was based on a meta-analysis that reported three levels of BCG efficacy: protection from TB disease and from infection (latent TB) and limiting the progression from infection to disease (i.e., from latent TB to active TB) [9]. Although other studies did not examine the VE relating to TB infection [7,38], we decided to incorporate these estimates because recent evidence has shown that the BCG vaccine not only prevents TB disease but also TB infection and its progression [8,10,39]. Another important driver of the analysis was the costs of vaccination. Although cost of vaccination only appeared once at the first year and seemed modest compared to the other healthcare costs, this variable was the second most influential one in the analysis. This implies that the price of vaccination plays an important role in reducing the overall of TB cost burden and decision-makers would be justified to pay more attention to implementing, optimizing, and sustaining TB vaccination programmes.

Our analysis was conservative, and health benefits relating to the BCG vaccination may therefore have been underestimated in the following way. First, we used a static Markov model and only considered the direct benefits of BCG vaccination on one closed birth cohort. This model may not have captured some of the indirect effects such as preventing TB transmission and immunity benefits. Inclusion of such effects within a dynamic-modelling approach would likely improve our cost-effectiveness estimates. Second, we did not consider BCG protection against more severe forms of TB disease in infants that showed a higher VE, for example, against meningeal TB and miliary TB (RR: 0.1; 95% CI: 0.01–0.77) [8]. Third, in our analysis, we did not consider the benefits of BCG on other mycobacterial infections, notably leprosy [40,41]. Indonesia ranks third among countries with the highest incidence of leprosy [42]. Therefore, a consideration of such outcomes may be warranted. Fourth, we did not consider the benefits of BCG vaccinations on all-cause, non-specific, child mortality [43]. Fifth, in our base-case analysis, we applied a conservative estimate of the duration of protection (10 years). When BCG is administered at birth, its protective effect lasts for approximately 10 to 15 years [8,10], although some studies suggest that protection may last longer [12,13]. The CIs associated with these estimates vary widely across different regions worldwide. In addition, we applied three different scenarios depicting the waning effect of the vaccine to assess plausible protection offered by the BCG vaccine.

Our findings indicated that even for a very conservative VE scenario, the vaccination strategy was still highly cost-effective in controlling TB disease. This conclusion may apply to other high TB-incidence countries. Our Markov model corroborated the finding of Trunz et al. [7], indicating that a universal BCG vaccination priced at US$2–3 is a highly cost-effective intervention for preventing severe childhood TB in high-incidence countries and where a high level of BCG coverage exists, notably within Southeast Asia, Africa, and the western Pacific region. However, our findings did not corroborate those of studies conducted in Japan and Taiwan, which are considered low-to-moderate TB-incidence countries. Notably, the study in Japan [38] revealed the high cost of universal BCG vaccination at US$35,950–175,862 for each prevented case, with the VE ranging from 40% to 80%. This cost exceeded the cost of treating one child with pulmonary TB (US$10,500). Similarly, in Taiwan, universal vaccination was predicted to have a lower health impact on the future TB burden [30].

Despite its robust results, our validated model still had some limitations. First, because no other high-quality studies have been conducted on BCG VE in Indonesia, VE estimates were derived from a meta-analysis using databases that did not specifically include high-incidence countries. Some studies have shown that the BCG VEs in high-incidence countries may differ from those in low-incidence settings. Consequently, we may have overestimated VE. Second, the age-stratified annual incidence was not directly accessible and was therefore calculated from two different sources. The number of new cases was reported from WHO population-based surveys, and the number of age-stratified groups was derived from a prediction demographic Indonesian dataset. The accuracy of these estimates is not known as Indonesia had the highest rate of TB underreported in 2017 [44]. Third, the estimates for treatment outcomes were also derived from a WHO dataset that was based on Indonesian surveillance data, a significant proportion of which were missing or lost to follow-up (more than 5% for DSTB treatment and 30% for MDRTB treatment). This issue may have introduced bias into the model. Given that the treated patients represented the same cost and outcome of TB patients assessed in the sensitivity analysis, we were able to address data loss at follow-up. Moreover, the reported treatment outcomes in this study (85.77% and 47.28% for DSTB and MDRTB, respectively) were very similar to estimates obtained in other studies for high TB-incidence countries, which were 85.7% and 47.3%, respectively [32,45]. Fourth, because of the lack of data, we simplified the analysis. For example, we did not differentiate the degrees of severity of TB disease in this model, instead using one estimate for the quality of life for active TB patients. We applied a higher QALY estimate for latent TB patients compared with that used for active TB patients. However, drawing on a study of latent and active TB patients in Canada and Indonesia [29,46,47], we applied a lower estimate relating to the healthy population. Additionally, in Indonesia, most drug-resistant TB patients are multidrug-resistant (MDRTB), with less than 10% being considered rifampicin mono-resistant (RRTB). Given the low incidence of RRTB, we assumed that both patterns of resistance had the same clinical and cost parameter values for treatment outcomes, mortality rates, and treatment costs. Fifth, in our modelling approach, we did not apply a stochastic individual-based modelling design. Notably, such a design is used if a lot of heterogeneity is present in the population investigated and detailed data at individual levels are available. We felt that, concerning TB, a birth cohort in Indonesia can be relatively well considered being homogeneous as well as that detailed data are currently still lacking in Indonesia. Therefore, we considered our cohort approach a valid one to estimate the costs and effects, with inclusion of an extensive uncertainty analysis as reflected in our Monte Carlo simulation. We do advise to consider other modelling types such as compartmental dynamic and individual-based models, if data on heterogeneity in geography, urbanization, and immunity would become available. These simplified approaches enabled the calculation of conservative estimates of efficacy and are unlikely to have had a marked impact on the results.

## 5. Conclusions

To conclude, our results support the continuation of the current BCG vaccination programme, especially in countries where the TB incidence remains high. Decision-makers in countries such as Indonesia with limited resources may consider our findings useful for reducing and controlling TB epidemics in their countries and supporting, optimizing, and sustaining TB vaccination programmes.

## Figures and Tables

**Table 1:** Input parameters used in the analysis.

Parameter	Base Case	Distribution (Interval) ^a^	References
Incidence (per 100,000 inhabitants)		Fixed	[19,20] calculated
Latent TB (pA)	27.27		
All forms of TB disease by age (pB)			
0–4	92.36		
5–14	65.66		
15–24	144.67		
25–34	176.95		
35–44	180.25		
45–54	230.03		
55–64	296.83		
>65	276.96		
RR disease in the latent TB population	21%	Log normal (14–30%)	[21]
Case Detection Rate	53%	Beta (48–58%)	[22]
Percentage of MDRTB (%)	2.74%	Beta (1.90–3.69%)	[22]
**Mortality Rate (Annually)**			
Healthy population	Age-stratified mortality rate		[23]
Case fatality rate	0.14	Beta (0.12–0.15)	[22]
Latent TB	0.03		[24]
**Untreated Patient Outcomes**			[25] calculated
Dead	0.11	Dirichlet	
Self-cured (pF)	0.03	Dirichlet	
**Treatment Outcomes (%)**			[22] calculated
DSTB treatment			
Successful (pE)	85.77%	Dirichlet (85–91%)	[22]
Failed (pD)	0.38%	Dirichlet	[22]
Dead	2.46%	Dirichlet	[22]
Lost to follow-up	5.38%	Dirichlet	[22]
MDRTB treatment			
Successful (pE)	47.28%	Dirichlet (47–69%)	[22]
Failed (pD)	3.71%	Dirichlet	[22]
Dead	16.17%	Dirichlet	[22]
Lost to follow-up	31.12%	Dirichlet	[22]
**Vaccine Efficacy**			
TB infection	27%	Beta (13–39%)	[9]
TB disease	71%	Beta (42–85%)	[9]
Progression from TB infection to disease	58%	Beta (23–77%)	[9]
**Costs (US$) in 2018**		
Vaccination cost	14	Gamma (±25%)	[26]
DSTB treatment	35	Gamma (±25%)	[27]
MDRTB treatment	1296	Gamma (±25%)	[27]
**Productivity Losses**			
DSTB (days)	25	Normal (±25%)	[6]
MDRTB (days)	102	Normal (±25%)	[6]
**Average Net Income per Day by Age (US$) in 2018**			[28]
15–24	3.94	Gamma (3.72–4.15)	
25–55	4.82	Gamma (4.70–4.95)	
55+	3.92	Gamma (3.72–4.12)	
**Utility**			
Healthy	1.00		assumed
Latent TB	0.82	Beta (0.80–0.85)	[29]
Active TB	0.68	Beta (0.65–0.72)	[29]
Treatment	0.68	Beta (0.65–0.72)	assumed
**Discount Rate (Costs and Utility)**	3%		[18]

Note: TB = tuberculosis, RR = relative risk, DSTB = drug-susceptible tuberculosis, MDRTB = multidrug-resistance TB. pA = probability of infection reflected as latent TB incidence, pB = probability of fast activation reflected as age-stratified active TB incidence, pC = probability of slow progression calculated by multiplying TB incidence with relative risk (RR) of developing TB disease in latent TB population, pD = probability of treatment failure, pE = probability of successful treatment, pF = probability of self-cure. ^a^ Interval reflected as the lower and upper value, we used 95% confidence intervals if applicable or ±25% deviations to the point of estimate in the base case. We applied specific distributions to the central estimates in the probabilistic sensitivity analysis.

**Table 2:** Results of the analysis of the base-case scenario for the vaccination strategy.

Variables	Healthcare Perspective			Societal Perspective		
	*Vaccination*	*No Vaccination*	*Incremental*	*Vaccination*	*No Vaccination*	*Incremental*
Total cost (US$ 2018)	65,876,510	11,167,980	54,708,530	79,232,212	28,203,661	51,028,551
Total QALY	140,558,261	140,069,668	488,592	140,558,261	140,069,668	488,592
ICER (US$ per QALY)			112			104
Total LYGs	306,314,453	305,235,474	1,078,979	306,314,453	305,235,474	1,078,979
Total new cases	529,358	579,071	49,713	529,358	579,071	49,713
Total deaths	2,509,157	2,516,755	7598	2,509,157	2,516,755	7598

Note: QALY = quality-adjusted life year, LYG = life year gained, ICER = incremental cost-effectiveness ratio.

## Supplementary Materials

The following are available online at https://www.mdpi.com/2076-393X/8/4/707/s1, Figure S1: Model internal validation, Figure S2: Model external validation title, Table S1: Stratified incidence by age, Table S2: Fitting the waning effect of vaccine effectiveness (VE) for scenario 1 and 2.
