# Supplementary material for: Cost-Effectiveness Analysis of BCG Vaccination against Tuberculosis in Indonesia: A Model-Based Study
Source: Vaccines (Basel). 2020 Nov 26;8(4):707. doi: 10.3390/vaccines8040707 (PMC7711585; doi:10.3390/vaccines8040707)
Supplement: Supplementary file 1 [file vaccines-08-00707-s001.pdf]

Supplementary Table S1: Stratified incidence by age

	Number of new and relapse cases[a]			Number of age group	
Age group	*male cases*	*female cases*	*total cases*	*population*[b]	*incidence*
0-4	11848	10355	22203	24039675	0,00092
5-14	16335	14406	30741	46819525	0,00066
15-24	31350	32695	64045	44268729	0,00145
25-34	41750	32327	74077	41864399	0,00177
35-44	41617	29166	70783	39269150	0,00180
45-54	44505	28835	73340	31883182	0,00230
55-64	39836	22321	62157	20940415	0,00297
>65	27761	13523	41284	14906304	0,00277

[a]source : World Health Organization, TB Burden Estimate for Indonesian settings in 2017. https://www.who.int/tb/country/data/download/en/

[b]source : Indonesia Population Projection in 2017, Indonesian National Statistics (Badan Pusat Statistik, BPS), 2013.

Supplementary Table S2: Fitting the waning effect of vaccine effectiveness (VE) for scenario 1 and 2.

Scenario 1 : we assumed that the vaccine effectiveness would wane linearly over a 20-year period. Accordingly, we estimated that the average protection would be comparable to that assumed in the base-case scenario.

Scenario 2 : we assumed that the vaccine's effectiveness would wane exponentially over time

Scenario 1

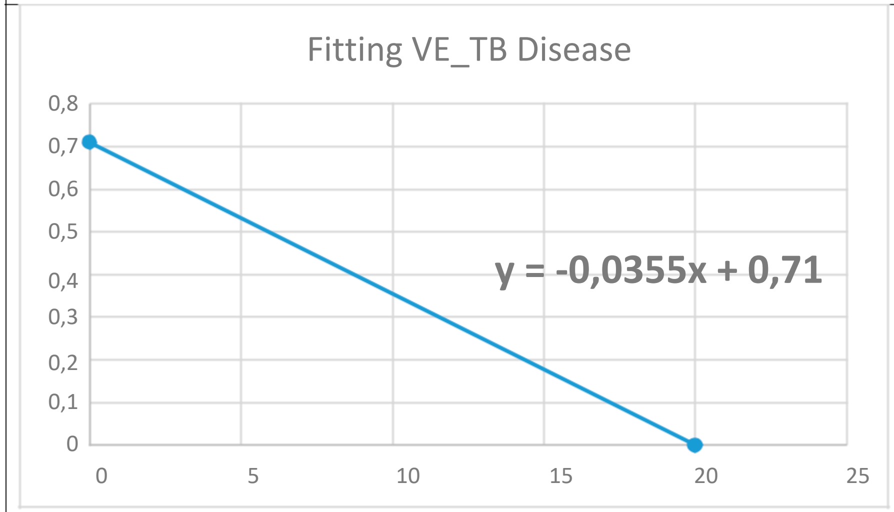

Scenario 2

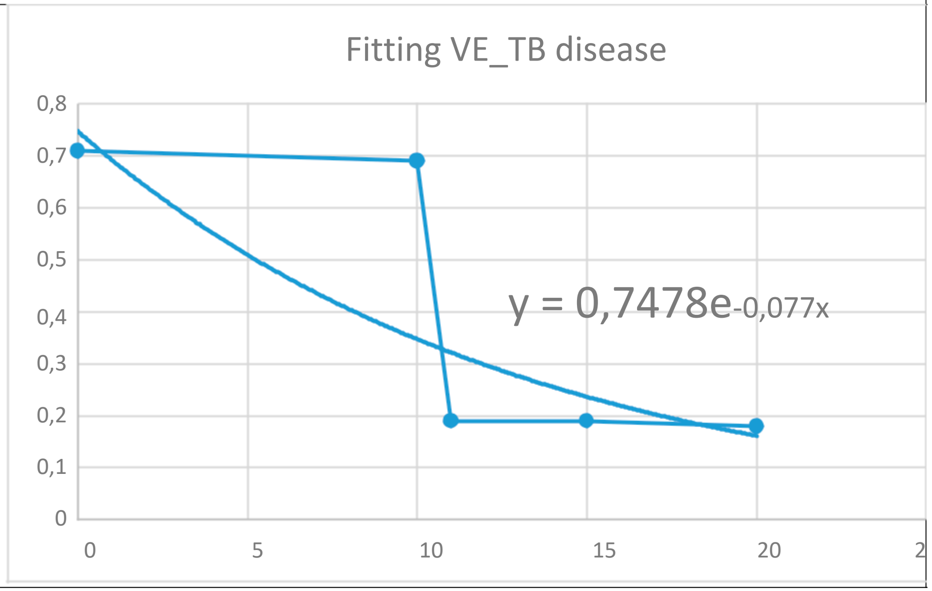

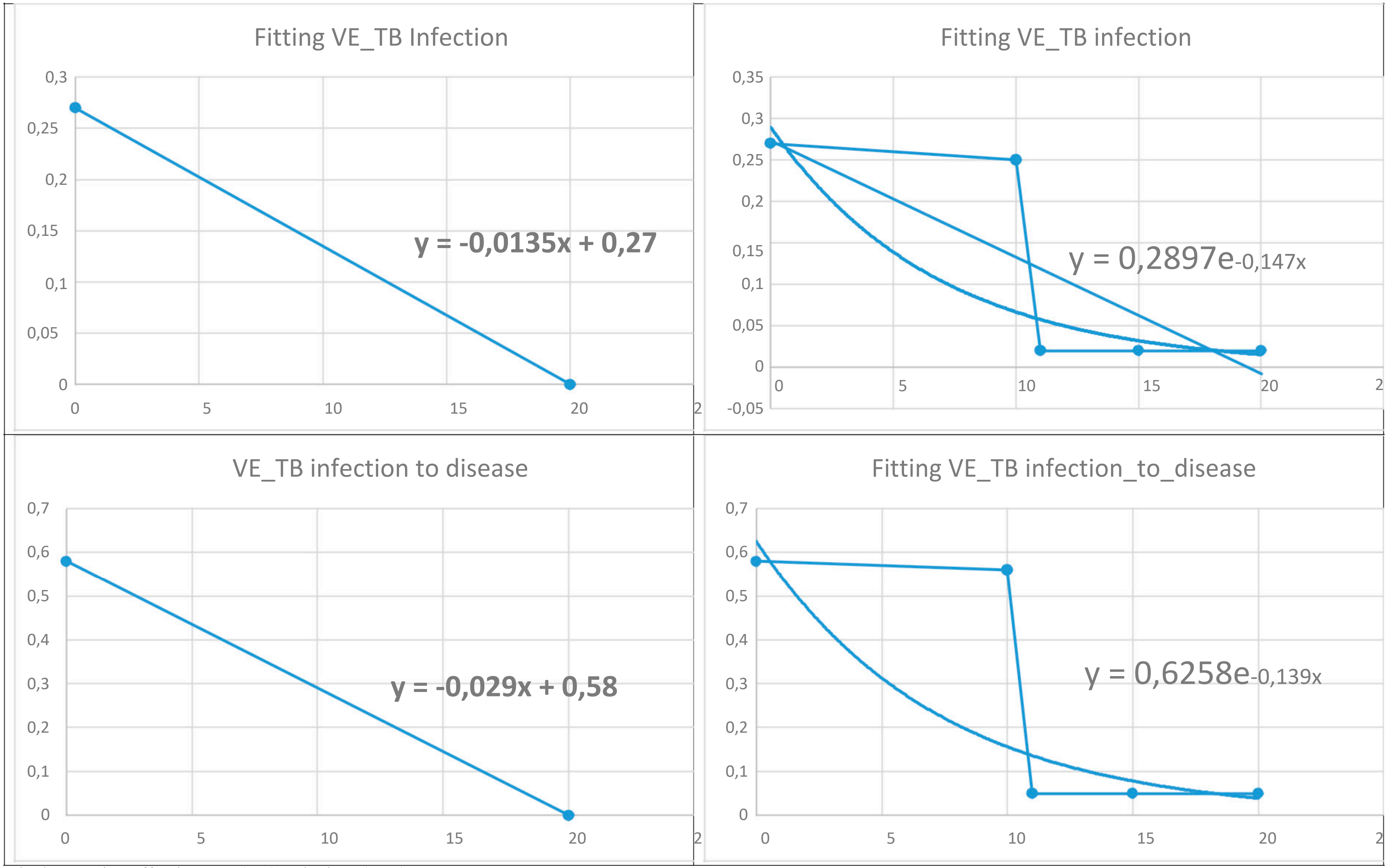

y is the vaccine effectiveness (VE), x is time (year)

Supplementary Figure S1: Model internal validation

We validated the model by comparing the TB incidence derived from the predicted simulation with that in the observed dataset used for the input parameters.

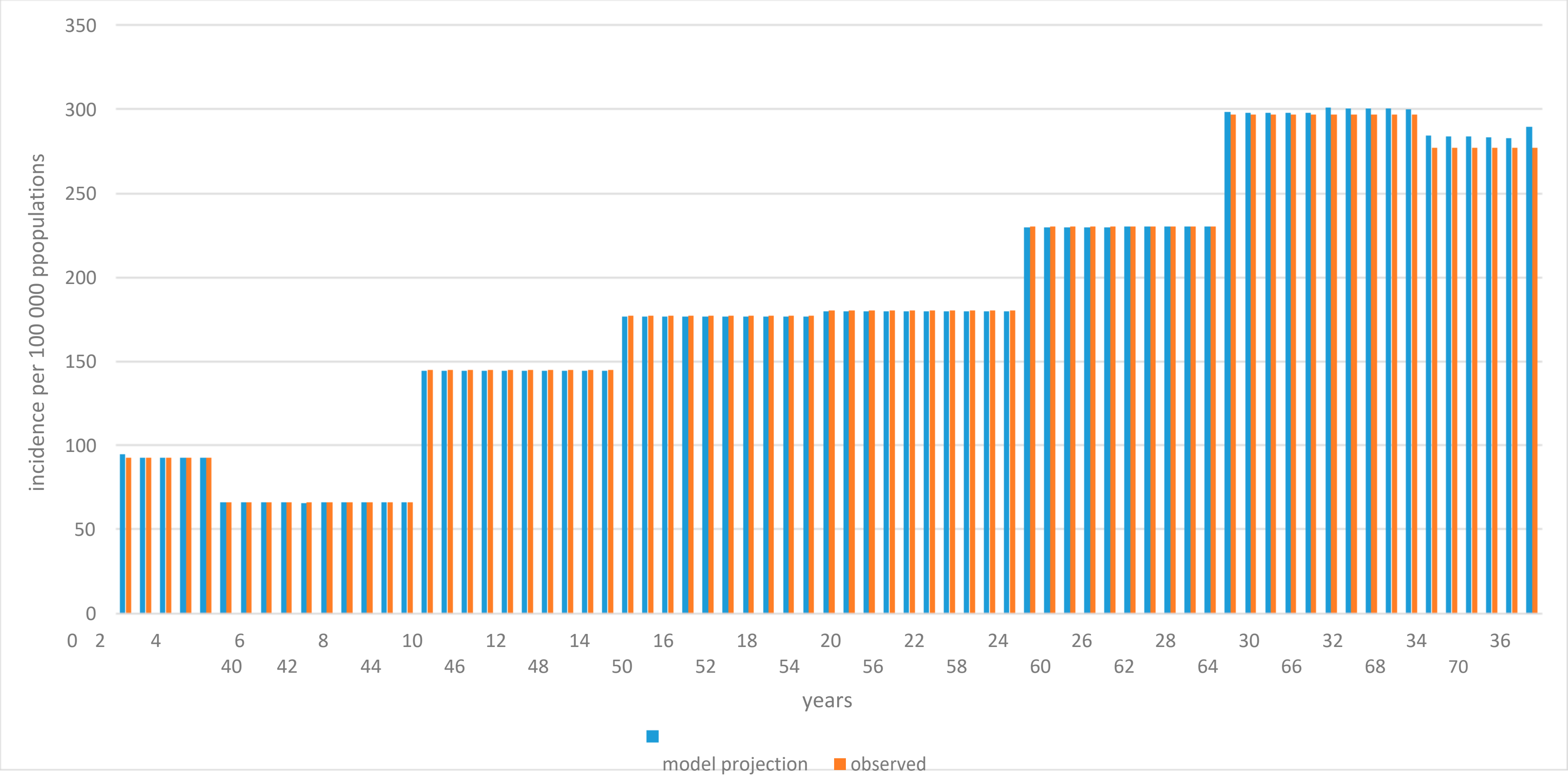

Supplementary Figure S2: Model external validation

We calibrated the model predicted TB prevalence on that obtained from local data in 2018.

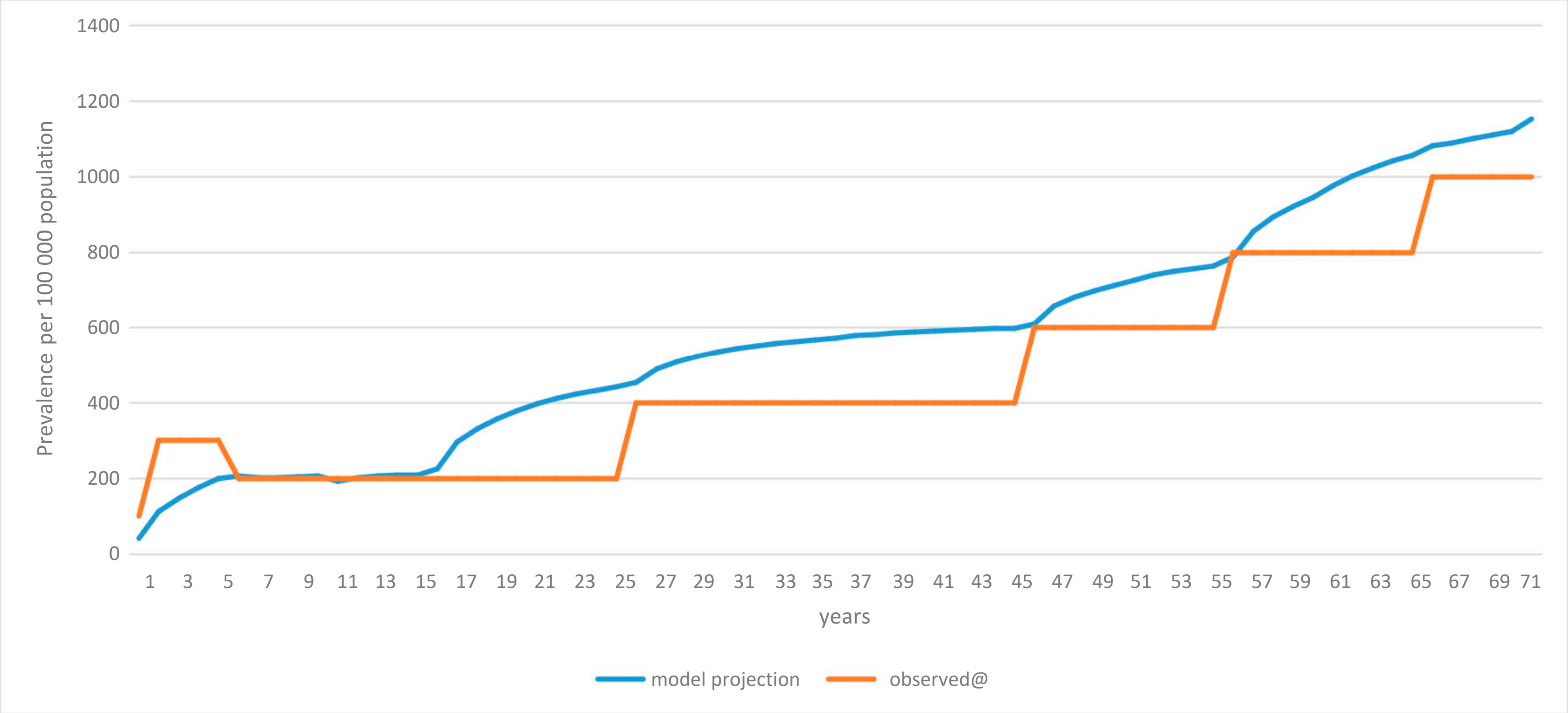

Source observed data : BALITBANGKES. Laporan Nasional RISKESDAS 2018 [Internet]. 2019 [cited 2019 Jun 13]. Available from: http://labmandat.litbang.depkes.go.id/images/download/laporan/RKD/2018/Laporan_Nasional_RKD2018_FINAL.pdf.
